# Case Series of Streptococcus pneumoniae Keratitis and Its Sequelae

**DOI:** 10.7759/cureus.73025

**Published:** 2024-11-04

**Authors:** Nadzirah Saffian, Qi Zhe Ngoo, Julieana Muhammed

**Affiliations:** 1 Department of Ophthalmology and Visual Sciences, School of Medical Sciences, Health Campus, Universiti Sains Malaysia, Kota Bharu, MYS; 2 Department of Ophthalmology, Hospital Tuanku Jaafar, Seremban, MYS

**Keywords:** antibiotics, cornea ulcer, malaysia, penetrating keratoplasty, perforation, streptococcus pneumonia

## Abstract

*Streptococcus pneumoniae* is a more prevalent cause of corneal ulcers in developing countries than in developed countries. We report five cases of corneal ulcers attributed to this organism with a literature review on bacterial cornea ulcers. Among the patients, four had underlying systemic illnesses while one was otherwise healthy. Four patients were presented with visual acuity worse than 6/60, and one patient had a visual acuity of 6/18. Two of the patients developed corneal perforation, which required tectonic penetrating keratoplasty. For visual outcome, two patients improved to visual acuities of 6/24 and 6/12, while three others had hand movement (HM) vision. All patients received broad-spectrum antibiotics, which were later adjusted based on culture sensitivity results. Notably, *S. pneumoniae* keratitis lacks specific ulcerative characteristics and can progress rapidly, frequently resulting in a guarded visual prognosis.

## Introduction

*Streptococcus pneumoniae *is a gram-positive diplococcus that produces alpha hemolysis. This bacterium is non-motile and does not form spores. It is part of the normal flora of the respiratory tract, nasal cavity, and skin. While* S. pneumoniae *is commonly associated with various infections, including pneumonia, meningitis, and otitis media, it can also play a role in the development of corneal ulcers. Corneal ulcers, also referred to as keratitis, are serious conditions characterized by the erosion of the corneal layer and, if not treated promptly, can lead to blindness.

In developing countries,* S. pneumoniae* appears to be a common pathogen responsible for bacterial corneal ulcers in contrast to developed countries. In South Africa and Madurai India, the prevalence of cornea ulcers caused by *S. pneumoniae* are 47.6 % and 44.3%, respectively [1,2]. Conversely, *Pseudomonas aeruginosa* is more frequently found in developed countries, largely due to the higher risk associated with contact lens use [3].

Virulence factors of *S. pneumoniae* are pneumolysin, IgA1 protease, polysaccharide capsule, and teichoic acid. According to a study, pneumolysin plays a significant role in the pathogenicity of cornea ulcers, causing considerable damage in a rabbit model of pneumococcal keratitis [4,5]. Pneumolysin binds to the cell membrane, forming pores that disrupt cellular integrity. This cytotoxic effect leads to cell lysis and tissue damage in both epithelium and stroma.

The diagnosis is typically made through clinical examination combined with microbial testing to identify the organism. Treatment involves topical antibiotics with supportive measures to promote healing and prevent complications. Surgical intervention might be required if complications develop.

In this paper, we present a case series of *S. pneumonia*e keratitis from a teaching hospital in West Malaysia along with a review of the related literature. This report aims to assist ophthalmologists in identifying risk factors and preventing the potentially devastating complications associated with *S. pneumoniae* keratitis.

## Case presentation

Case 1

**Figure 1:**
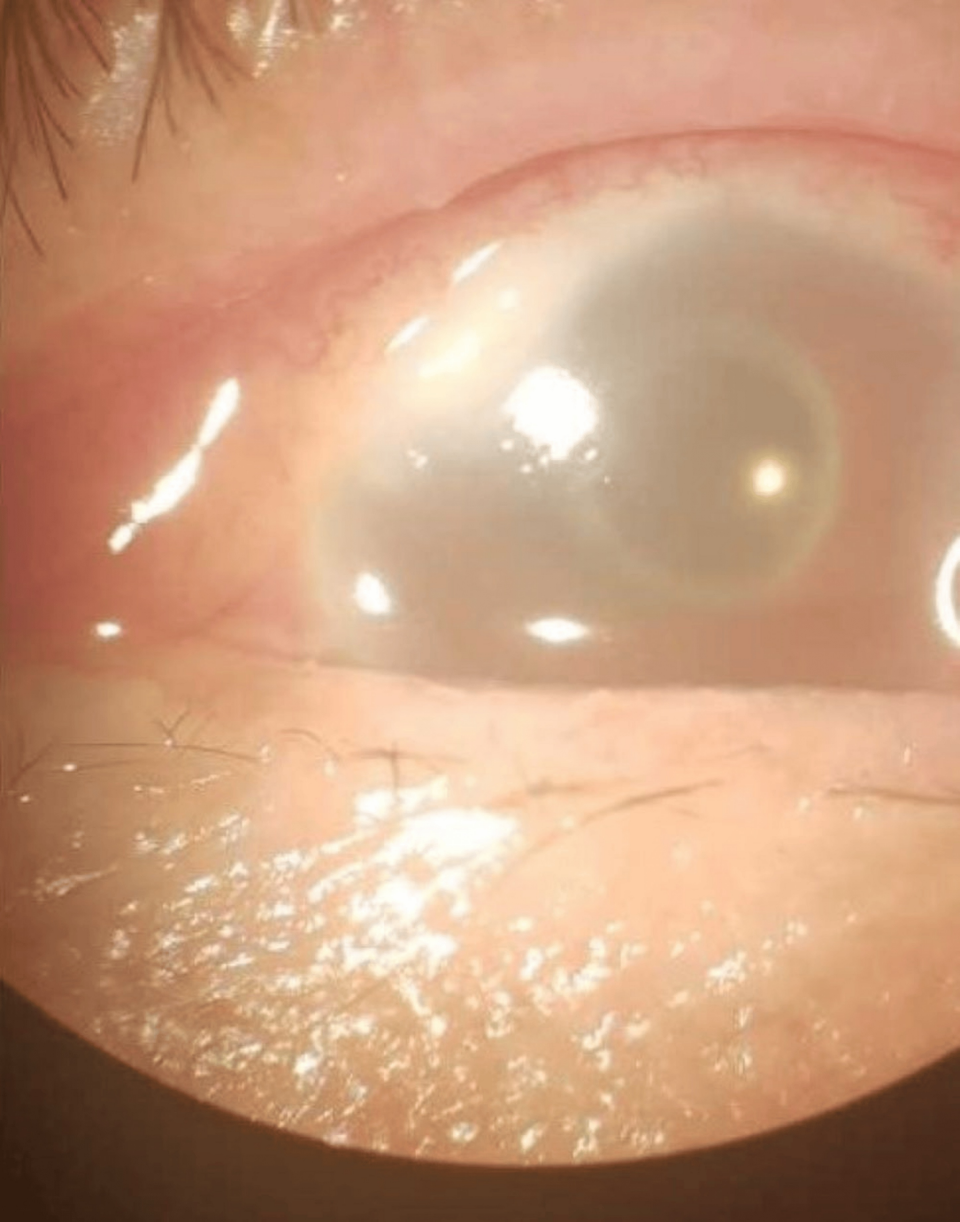
Case 1 Superior corneal stromal infiltrate originates from the site of the surgical wound.

A 77-year-old male with underlying diabetes mellitus presented with acute pain and redness in the right eye. He had a history of phacoemulsification surgery performed five years prior, which was uneventful. Upon examination, he demonstrated a visual acuity of hand movements (HM) and a peripheral, dense superior corneal stromal infiltrate originating from the site of the surgical wound (Figure 1). There was no hypopyon. Culture and sensitivity (C&S) testing identified *S. pneumoniae,* which was sensitive to fluoroquinolone, penicillin, and tetracycline antibiotics. Consequently, intensive treatment was initiated with vancomycin 0.2% and moxifloxacin 0.5% eyedrops, alongside intravenous vancomycin at a dosage of 1 g twice daily, as the pathogen was sensitive to both medications. Despite this treatment, the ulcer progressed to thinning and perforation, necessitating tectonic penetrating keratoplasty. Subsequent follow-up revealed an intact corneal graft with no signs of recurrence; however, the patient's visual acuity remained unchanged.

Case 2

**Figure 2:**
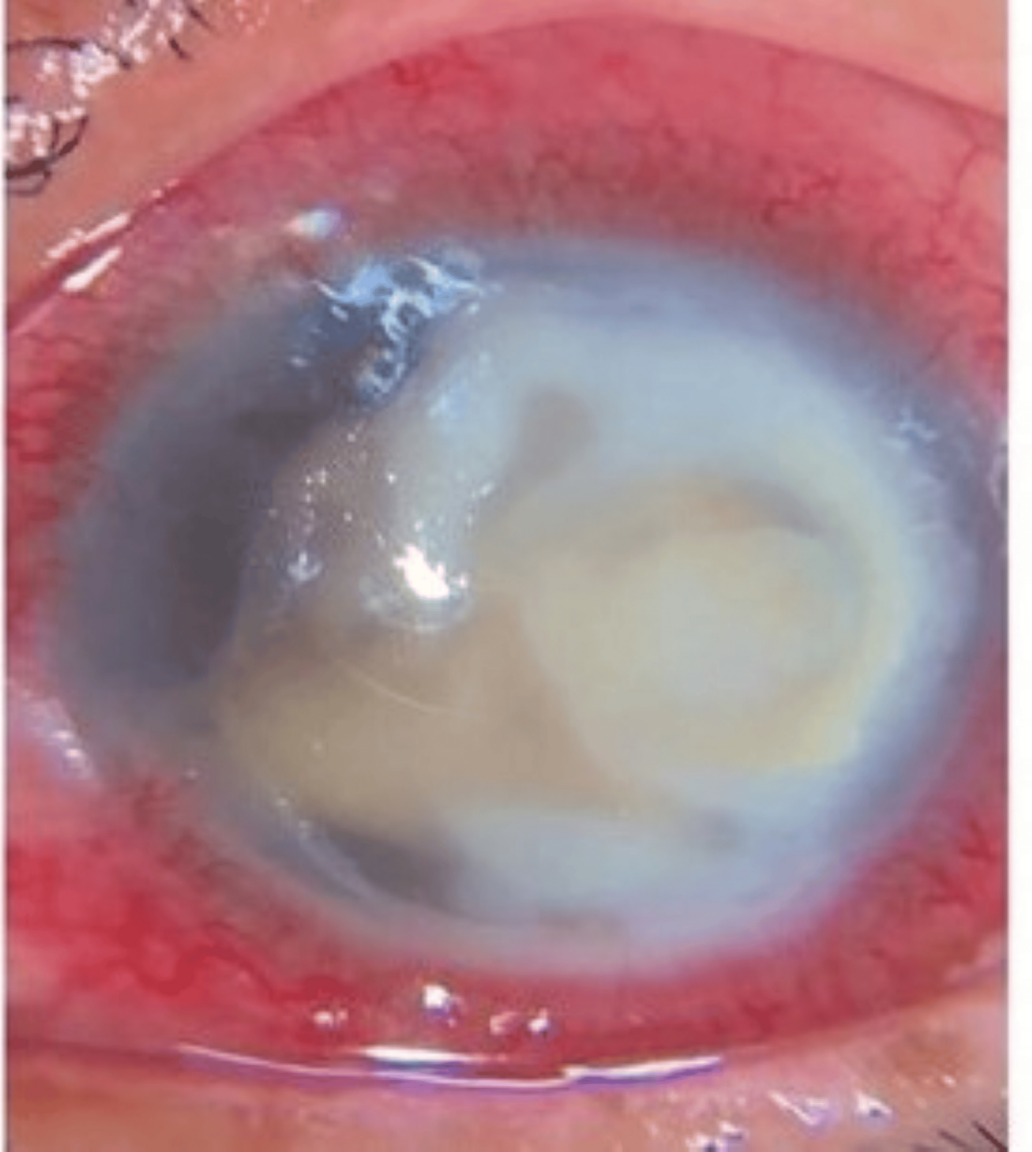
Case 2 A peripheral, C-shaped temporal dense corneal stromal infiltrate accompanied by an endothelial plaque and streaks of hypopyon.

A 57-year-old man with underlying diabetes mellitus presented with pain and redness in his right eye for two days following a history of being struck by a stone. Ocular examination revealed a visual acuity of HM and a peripheral, c-shaped temporal dense corneal stromal infiltrate accompanied by endothelial plaques and streaks of hypopyon (Figure 2). C&S testing identified *S. pneumoniae*, which was sensitive to fluoroquinolone and cephalosporin. Despite intensive treatment with ceftazidime 5% and moxifloxacin 0.5%, the infiltrate remained unchanged, and the patient's visual acuity deteriorated to no light perception. Given the progressive thinning and perforation of the cornea despite maximum medical therapy, tectonic penetrating keratoplasty was performed, resulting in no recurrence of the condition.

Case 3

A 53-year-old healthy man presented with acute pain and redness in his right eye for four days following an incident in which a foreign body entered his eye while cutting grass. Upon examination, his visual acuity was 6/18. The ocular examination revealed a dense, round infiltrate at the center of the cornea, measuring 5.0x5.0 mm, without the presence of hypopyon. Given the nature of the foreign body, the patient was initially treated for fungal keratitis with intensive topical moxifloxacin 0.5%, vancomycin 0.2%, and amphotericin B 0.15%, supplemented with oral fluconazole at a dosage of 200 mg once daily. C&S yielded *S. pneumoniae, *which was sensitive to fluoroquinolone and cephalosphorine. Consequently, the vancomycin eye drops were changed to ceftazidime 5%. Due to slow healing, the patient required long-term treatment. After six months of continuous therapy, the ulcer healed, resulting in central corneal scarring and the development of pseudopterygium. Ultimately, the visual acuity in the right eye remained at 6/24, with pinhole vision improved to 6/18.

Case 4

A 70-year-old woman with a history of diabetes mellitus and hypertension presented to the emergency department with right eye redness and decreased vision for four days. She had constant eye irritation, leading to excessive rubbing, but had no previous eye surgeries. Upon examination, her visual acuity in the right eye was counting fingers (CF), while the left eye was 6/9. A round, well-defined infiltrate measuring 2 mm x 2 mm was observed in the stroma near the peri-pupillary margin, accompanied by hypopyon in the anterior chamber. Treatment with 5% ceftazidime and 0.5% moxifloxacin eye drops was initiated. Subsequent C&S testing yielded *S. pneumoniae*, which was sensitive to current eye drops. After three weeks of intensive treatment, the medications were gradually tapered as the corneal infiltrate improved, leaving a scar in the peri-pupillary area. Sequential follow-ups revealed no recurrence, and visual acuity in the right eye improved to 6/12, with no improvement with the pinhole.

Case 5

**Figure 3:**
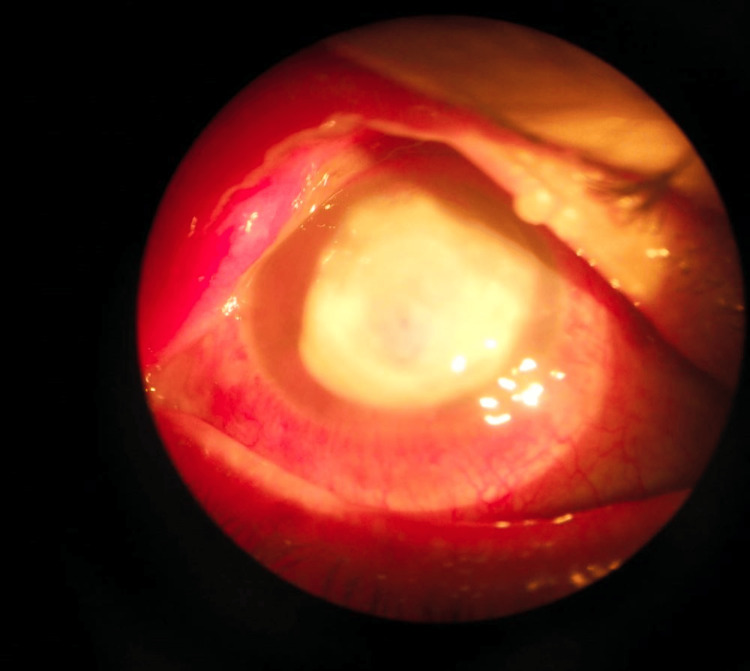
Case 5 A dense, round infiltrate with a rolled border occupies almost the entire cornea, with a central area of thinning.

A 44-year-old man with underlying human immunodeficiency virus (HIV) and hepatitis B presented with sudden reduced vision, pain, and discharge for three days. No history of eye trauma or eye surgery. On examination, the right visual acuity was HM, subtotal dense round infiltrate 7x6 mm, with a rolled border occupying almost the whole cornea, the central area of thinning, with the presence of hypopyon in the anterior chamber (Figure 3). Intensive treatment with empirical antibiotic moxifloxacin 0.5% and vancomycin 0.2% was given with additional oral doxycycline 100 mg twice daily. After four days C&S yielded *S. pneumoniae*. In view of the slow healing of the ulcer, with an immunocompromised state, oral ciprofloxacin 750 mg twice daily was added after one week of treatment. After a month of treatment in the ward, the infiltrate showed signs of improvement as it was less dense and smaller with a measurement of 5.0x6.0 mm. Therefore, the patient was discharged with tapering medication. However, the patient defaulted to follow-up.

## Discussion

**Figure 4:**
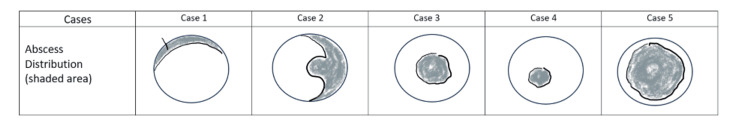
Diagram illustrates the distribution of ulcers in patients

Corneal ulcers caused by *S. pneumoniae* are relatively uncommon in Malaysia [6]. In this case series, we report on five patients: four males and one female. Four of the patients had significant systemic illnesses: three with long-standing diabetes mellitus and one with HIV. One patient did not have any systemic illness. Four patients had identifiable ocular risk factors. One had undergone cataract extraction five years prior, while two had a history of eye trauma involving foreign bodies penetrating the cornea, and one had a history of chronic dry eyes. All cases are summarized in Table 1. Figure 4 illustrates the distribution of ulcers in the patients.

**Table 1:** Demographics, clinical presentation, treatment, and outcome of the patients HM, hand movement; CF, counting finger

Case	Case 1	Case 2	Case 3	Case 4	Case 5
Age	77	57	53	70	44
Gender	Male	Male	Male	Female	Male
Ocular risk factors	Cataract surgery	Ocular trauma	Ocular trauma	Ocular surface disease	Nil
Systemic illness	Diabetes mellitus	Diabetes mellitus	Nil	Diabetes mellitus	HIV, hepatitis B
Vision on presentation	HM	HM	6/18	CF	HM
Treatment	Vancomycin and moxifloxacin	Ceftazidime and moxifloxacin	Moxifloxacin, vancomycin, and amphotericin B	Ceftazidime and moxifloxacin	Moxifloxacin and vancomycin
Complicated with perforation	Yes	Nil	Nil	Nil	Nil
Tectonic keratoplasty	Yes	Yes	No	No	No
Final visual outcome	HM	HM	6/18	6/12	HM

Few studies reported a higher prevalence of corneal ulcers in males compared to females [2,6,7]. This is due to more outdoor activities among males, increased occupational hazards such as agricultural workers or laborers, and delayed hospital visits and seeking treatment in males. On the contrary, Suwal et al. reported that in the Nepali population, females were more frequently affected by corneal ulcers than males. This may be due to the greater involvement of females in agricultural and household activities in this region [8]. However, in developed countries, younger female patients are more commonly observed to develop contact lens-related corneal ulcers [7].

*S. pneumoniae* corneal ulcers are usually seen more frequently in the elderly than in younger age groups, this is consistent with our cases, where all of the patients were over 40 years old. Advanced age, ocular surface disease (OSD), past ocular surgery, preexisting ocular morbidities, and systemic illnesses all increase the overall risk of visual morbidity in older age groups [6,8,9]. *S. pneumoniae *keratitis is frequently observed in patients with a history of ocular surgery. The risk factors include loose sutures, inadequate ocular surface conditions, and graft decompensation in individuals who have undergone penetrating keratoplasty [10]. Meanwhile, in younger patients, *P. aeruginosa* is more commonly associated with contact lens-related corneal ulcers [6,8].

The clinical diagnosis of *S. pneumoniae* corneal ulcers can be challenging due to their variable presentation. C&S testing from corneal scrapings remains the gold standard for identifying the organism. The characteristics of these lesions may vary based on factors such as symptom duration, underlying systemic conditions, and the pre-infection status of the cornea. Typically, *S. pneumoniae* corneal ulcers present as distinct abscess-like lesions, often described as deep, oval ulcers located centrally on the cornea, frequently accompanied by hypopyon and corneal thinning. In cases of* S. pneumoniae* keratitis following cataract surgery, the condition often presents as a peripheral corneal infiltrate, commonly associated with surgical sutures [9]. These findings are similar to our cases, where a patient with a history of cataract extraction exhibited an infiltrate on the periphery near the surgical suture. In contrast, other patients presented with deep oval infiltrates located centrally on the cornea.

The primary goal of antibiotic treatment for corneal ulcers is to inhibit bacterial growth and promote healthy epithelial healing. Fluoroquinolone monotherapy has been shown to be as effective as combination therapy while exhibiting lower toxicity. In this case series, all patients received empirical treatment with moxifloxacin (fluoroquinolone group) alongside either vancomycin or ceftazidime (cephalosporin group). Moxifloxacin, a newer generation fluoroquinolone, is commonly utilized for the treatment of corneal ulcers due to its effective action against both gram-positive and gram-negative bacteria [3,8]. Furthermore, it has demonstrated lower resistance rates to *Staphylococcus aureus* and *P. aeruginosa* in the USA and India, respectively. Notably, moxifloxacin has been reported not to cause precipitates on the cornea, in contrast to ciprofloxacin [3]. The role of corticosteroids in the treatment of bacterial corneal ulcers remains controversial. According to one study, topical steroids do not appear to have negative side effects or provide significant benefits when used in conjunction with topical antibiotics for the treatment of corneal ulcers [10].

Penetrating keratoplasty is indicated as the surgical treatment for corneal perforation when the condition does not improve despite maximum medical treatment or when impending perforation is anticipated. Studies have identified several risk factors associated with poor outcomes that may necessitate penetrating keratoplasty, including advanced age, history of ocular surgery, prior use of topical steroids, delayed referral to a corneal specialist, poor vision at presentation, and the presence of large, centrally located ulcers [10]. In our cases, two patients who underwent penetrating keratoplasty presented with poor vision (HM) and had large corneal ulcers, and one of them had a history of prior ocular surgery.

Final visual outcomes were assessed during the patient's last clinic visits and varied from HM to a best corrected visual acuity (BCVA) of 6/12. The two patients who experienced corneal perforation had a BCVA of HM. In contrast, the remaining two patients, whose corneal ulcers healed with scarring, achieved BCVA of 6/24 and 6/12, respectively. Another patient who defaulted on follow-up had a BCVA that remained at HM.

The vision at the presentation, as well as the size and location of the ulcer, had significant effects on the final visual outcomes for the patients. Large and centrally located ulcers obstruct the visual axis; when an ulcer heals with a scar, it can block the visual axis and result in poor vision. Omar et al. also reported that age is a significant factor in determining visual outcomes. Elderly patients often have more systemic illnesses and ocular surface diseases that may impair the healing of ulcers [6].

## Conclusions

*S. pneumoniae* is not a common organism associated with microbial keratitis. However, it is more frequently observed in elderly patients compared to younger individuals, particularly among those with multiple ocular morbidities, history of ocular surgery, and underlying systemic illnesses. Prompt treatment, especially with fluoroquinolone and other appropriate antibiotics, is crucial to prevent complications. The prognosis of *S. pneumoniae* keratitis varies depending on the duration of symptoms before treatment initiation, as well as the patient's ocular and systemic comorbidities.
